# From Pain Relief to Multidimensional Outcomes: A Structured Narrative Review of Success Language in a PubMed/MEDLINE Spinal Cord Stimulation Corpus

**DOI:** 10.3390/jcm15135216

**Published:** 2026-07-03

**Authors:** Jakub Wiśniewski, Mateusz Szczupak, Paweł Jan Winklewski, Anna Barbara Marcinkowska

**Affiliations:** 1 Department of Neurosurgery, Nicolaus Copernicus Hospital, 80-803 Gdansk, Poland; 2 Department of Anaesthesiology and Intensive Therapy, Nicolaus Copernicus Hospital, 80-803 Gdansk, Poland; mszczupak@copernicus.gda.pl; 3 Department of Neurophysiology, Neuropsychology and Neuroinformatics, Medical University of Gdansk, 80-210 Gdansk, Poland; pawel.winklewski@gumed.edu.pl; 4 2nd Department of Radiology, Medical University of Gdansk, 80-210 Gdansk, Poland

**Keywords:** chronic pain, neuromodulation, outcome assessment, patient-reported outcome measures, spinal cord stimulation, therapeutic success, treatment outcome

## Abstract

**Background/Objectives:** Spinal cord stimulation (SCS) is usually evaluated through pain relief, yet the published language of therapeutic success is broader. This structured narrative review examined success language in the SCS literature to map how outcome terminology has accumulated across domains. **Methods**: A PubMed/MEDLINE corpus was retrieved on 23 May 2026 using the strategy (“Spinal Cord Stimulation”[Mesh] OR “Spinal Cord Stimulation”[Title/Abstract]). The search returned 5719 records from 1961 to 2026. Title-and-abstract screening was performed independently by two authors, with pre-consensus agreement of 99.77% (Cohen’s κ = 0.995). After screening, 3687 records were retained for thematic narrative synthesis. **Results**: Pain relief or analgesic response appeared in 3550 retained records (96.3%) and remained the dominant outcome language across decades. Additional domains accumulated around it: function, quality of life, and sleep (72.5%); durability (55.5%); complications and device burden (51.0%); trial stimulation (44.1%); patient selection (40.5%); paresthesia coverage (29.9%); patient preference (24.5%); economic or occupational outcomes (22.8%); stimulation paradigm (21.9%); and medication use (21.3%). Physiological-feedback language was separated into mechanism-related language, objective monitoring, and evoked compound action potential (ECAP)-controlled closed-loop feedback. **Conclusions**: Within this corpus, SCS success emerged as a layered concept: analgesia remained central but was increasingly reported alongside other outcome domains rather than standing as the sole reported marker of therapeutic success. Importantly, this review maps the published language of outcome reporting, not clinical practice, payer behaviour, or any formal redefinition of treatment success; the findings should not be read as evidence that SCS success is now uniformly defined in multidimensional terms, and this scope is essential when interpreting the findings.

## 1. Introduction

Spinal cord stimulation (SCS) is often introduced through hardware: leads, pulse generators, programming contacts, waveforms, rechargeable batteries, and closed-loop devices. There is nothing wrong with that account, but a device chronology leaves aside a more clinical question: what has the literature considered a successful result? Early PubMed-indexed reports already treated success as unstable. Pain relief mattered, yet clinicians read it through trialability, paresthesia, anatomical targeting, and the patient’s response to stimulation before permanent implantation [1,2].

Paresthesia provided the early field with a map. Doerr et al. related pain perception to induced paresthesia after long-term dorsal column stimulation, and Holsheimer et al. later treated paresthesia steering, coverage, thresholds, and therapeutic range as performance measures in their own right [3,4]. That older sensory–technical model made analgesia clinically legible, but it also reached its limits once investigators began evaluating SCS through longer follow-up, comparative studies, psychological selection, device burden, and healthcare costs [5,6,7,8,9,10,11,12,13].

Pain relief still anchors the corpus. In the dataset analysed here, pain relief or analgesic response appeared in 3550 of 3687 retained records. What requires explanation is why so many other outcome domains accumulated around it: function, quality of life, sleep, medication use, revision, explantation, work status, cost, patient preference, stimulation paradigm, objective monitoring, and physiological feedback. The literature did not abandon analgesia; it made analgesia share the evidentiary space.

Several shifts made that expansion difficult to avoid. Randomised comparisons with reoperation and conventional management required outcomes beyond immediate pain change [6,9,10,11,14,15]. Long-term studies made durability visible [5,11,12,13,16,17]. Psychological and prognostic studies reframed success as a selection problem, asking not only whether SCS works but which patient is likely to benefit [7,14,15,18]. The patient’s account also became harder to compress into a responder threshold, and qualitative and patient-perspective studies brought expectation, satisfaction, recovery burden, and preference into the evaluation of SCS pathways [18,19]. Sleep emerged as an outcome in its own right [20,21], and medication studies redefined success as a change in therapeutic dependency rather than pain intensity alone [22,23].

Stimulation itself also became a more explicit part of the outcome claim. High-frequency, burst, subperception, and closed-loop paradigms shifted attention from whether stimulation was present to how clinicians delivered it and how patients perceived, preferred, and sustained it [16,24,25,26,27,28,29]. The mechanism-related literature asked how SCS may produce analgesia [30,31,32]. Objective monitoring introduced external or physiological correlates of treatment effect [33]. ECAP-controlled closed-loop stimulation added a narrower device-mediated claim, with stimulation adjusted according to recorded neural activation [21,34].

This structured narrative review examines the changing language of success in a PubMed/MEDLINE SCS corpus retrieved with a predefined search strategy. It does not ask whether one waveform outperforms another, nor does it attempt a pooled estimate of treatment effect. Earlier narrative reviews of SCS have focused primarily on mechanisms, indications, and clinical efficacy [35]. The present review addresses a narrower and distinct question: how has the published language of therapeutic success itself has changed over time? The unit of analysis is the outcome concept visible in titles, abstracts, and PubMed metadata. The claim is bounded; the review maps published outcome language within this corpus, not uniform clinical practice across centres, payers, countries, or device platforms.

## 2. Materials and Methods

### 2.1. Study Design

This article was designed as a structured narrative review supported by title-and-abstract mapping of a PubMed/MEDLINE corpus. Its purpose is to examine how published outcome language has represented therapeutic success in SCS over time; it does not estimate pooled treatment effects, compare stimulation paradigms quantitatively, or perform formal evidence grading, and corpus mapping served as a structuring device rather than a substitute for a systematic-review methodology. The review shares procedural features with scoping reviews—a predefined search strategy, structured screening, and quantitative domain mapping—but differs in analytic target rather than technique. While a scoping review characterises the size, range, and nature of an evidence base [36], the present review treats the published vocabulary of success as its object of study and asks how that vocabulary has changed over time. The quantitative metrics reported here therefore index the reliability of thematic classification, not the coverage or completeness of an evidence map. Accordingly, we follow the conventions of a structured narrative review rather than PRISMA-ScR, did not perform formal evidence mapping or risk-of-bias assessment, and regard this work as complementary to, rather than a replacement for, a formal scoping review of the same field.

### 2.2. Data Source and Corpus Mapping

We generated a PubMed/MEDLINE corpus on 23 May 2026 using the NCBI Entrez utilities through Biopython. The search strategy was (“Spinal Cord Stimulation”[Mesh] OR “Spinal Cord Stimulation”[Title/Abstract]) and retrieved 5719 records spanning 1961–2026. The dataset represents the complete corpus retrievable with the predefined strategy at the time of export, not an unrestricted search of all biomedical, engineering, regulatory, or grey-literature sources. Extracted fields included PMID, DOI, title, abstract, journal title, publication year, authors, affiliations, MeSH terms, keywords, and publication type. The retrieval script is provided as Supplementary File S2, and the retrieved PubMed/MEDLINE CSV corpus is provided as Supplementary File S1.

### 2.3. Screening and Thematic Charting

All 5719 records were screened at the title-and-abstract level. Abstracts were available for 5144 records; for the remaining 575 records without abstracts, we used the title and available PubMed metadata. After structured screening and independent review by two authors, 3687 records were retained for thematic narrative synthesis, and 2032 were not retained. Retained records spanned 1975–2026. We retained records that concerned SCS and addressed one or more domains relevant to how therapeutic success has been defined, measured, or redefined. We excluded records when SCS was mentioned only incidentally; when the primary focus was non-pain rehabilitation, locomotor recovery, or motor restoration without pain outcome relevance; when the record was preclinical or animal-based without clinical outcome relevance; or when the available information was insufficient for thematic classification. The title-and-abstract classification output is provided as Supplementary File S3.

### 2.4. Reviewer Agreement and Adjudication

Two authors independently reviewed all 5719 AI-assisted screening decisions. Reviewer 1 modified the AI-assisted classification for 4 records and Reviewer 2 independently modified 9 different records, with no overlap between the two sets. Here Cohen’s κ = 0.995 refers to agreement between the two human reviewers, not between a reviewer and the AI output; the 4 and 9 records modified by the two reviewers together formed the 13 human–human discordances. AI-to-Reviewer 1 agreement was 99.93% (5715/5719 records); AI-to-Reviewer 2 agreement was 99.84% (5710/5719 records); and pre-consensus human-to-human agreement was 99.77% (5706/5719 records), with Cohen’s κ = 0.995. The high agreement reflects the predominantly binary nature of the retained/not-retained decision for most records rather than an absence of methodological complexity. All 13 discordant records were reviewed at the full-text level and retained after consensus discussion. We secondarily disaggregated the broad physiological-feedback category into three non-mutually exclusive subcategories: mechanism-related language (*n* = 1235, 33.5%), objective monitoring (*n* = 399, 10.8%), and ECAP-controlled closed-loop feedback (*n* = 144, 3.9%). The reviewer-level verification and adjudication file is provided as Supplementary File S4.

### 2.5. Language-Model Assistance

During the preparation of this review, we used ChatGPT GPT-5.5 Thinking (OpenAI) to assist with title-and-abstract screening, thematic organisation, methodological drafting, and language editing. The authors supplied the source dataset, search strategy, screening criteria, and prompts. The model was instructed to use only the uploaded PubMed CSV dataset and not to infer missing bibliographic details or full-text claims. AI-assisted screening output served as an initial structured classification for human verification. Final screening decisions, interpretive categorisation, and manuscript claims remained the responsibility of the authors. No manuscript text, references, or scientific conclusions were generated autonomously by the model. To assess potential algorithmic under-classification of older records characterised by non-standardised outcome language, we examined the pre-2000 subset of the corpus post hoc. All 611 pre-2000 records (279 retained, 332 not retained) were independently verified by both human reviewers under the same workflow applied to the full corpus. Of the 332 pre-2000 records not retained, 6 (1.8%) cited insufficient title-and-abstract information as the exclusion reason; the remainder had clearly classifiable exclusion criteria including non-pain rehabilitation or motor-function focus (*n* = 91), preclinical or animal-only records without clinical outcome relevance (*n* = 91), lower-centrality records (*n* = 108), no identifiable SCS focus in available metadata (*n* = 10), and no relevant success domain identifiable at the title-and-abstract level (*n* = 26). Of the 83 pre-2000 records without available abstracts, 9 were retained for thematic synthesis on the basis of title and PubMed metadata alone, confirming that title-level information was sufficient for the binary retention decision. These data do not indicate systematic under-retention attributable to non-standardised historical idioms, although a dedicated independent re-review of the pre-2000 subset was not performed.

### 2.6. Synthesis

**Figure 1:**
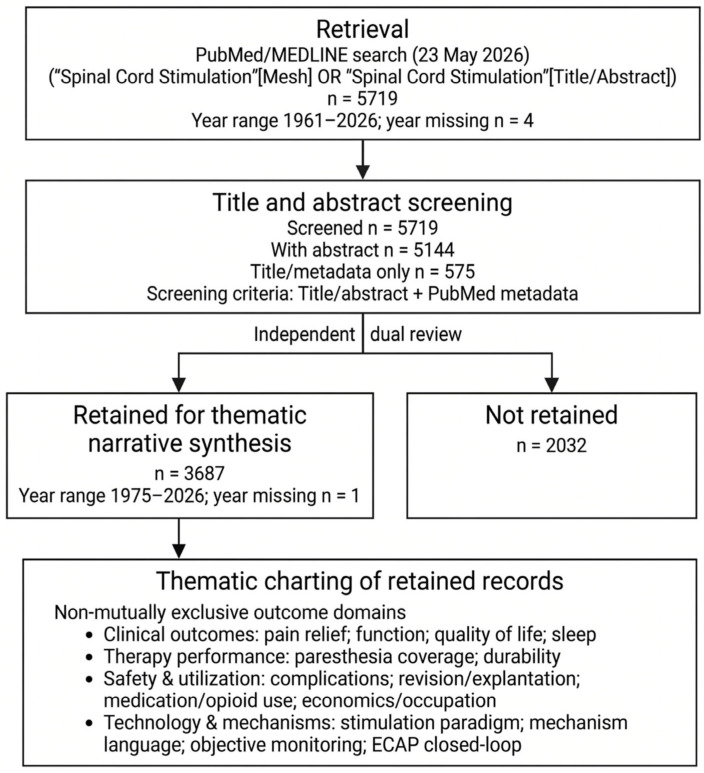
Flow of PubMed/MEDLINE corpus retrieval and thematic retention. A PubMed/MEDLINE title-and-abstract corpus was generated on 23 May 2026 using a predefined search strategy (“Spinal Cord Stimulation”[Mesh] OR “Spinal Cord Stimulation”[Title/Abstract]). The search retrieved 5719 records spanning 1961–2026; four records lacked a usable publication-year field. All records were screened at the title-and-abstract level; 5144 contained abstracts and 575 were assessed using the title and available PubMed metadata only. After structured screening and independent review by two authors (Cohen’s kappa = 0.995), 3687 records were retained for thematic narrative synthesis and 2032 records were not retained. Retained records spanned 1975–2026. Outcome domains were assigned using a multi-label approach. This diagram describes corpus construction for a structured narrative review; it is not a PRISMA flow diagram.

The synthesis was organised narratively around overlapping outcome domains, and the unit of analysis was the outcome concept. We performed no meta-analysis, risk-of-bias assessment, or formal evidence grading. Temporal patterns were interpreted as changes in how the retrieved corpus represented SCS success, not as direct evidence of uniform changes in clinical practice. We restricted quantitative temporal comparisons to decades with at least 200 retained records to reduce instability from small early denominators. Domains were assigned using a multi-label scheme and were not mutually exclusive, so a single record could contribute to several domains at once. For example, a long-term randomised trial reporting sustained analgesia together with improved quality of life and reduced opioid use would be charted simultaneously under pain relief/analgesic response, function/quality of life/sleep, durability/long-term follow-up, and medication/opioid use. Figure 1 illustrates the corpus construction process. A compact graphical summary of the overall review workflow (retrieval, title-and-abstract screening, thematic charting, and narrative synthesis) is additionally provided in the Supplementary Materials (Supplementary File S6).

## 3. Results

### 3.1. Corpus Overview

The search retrieved 5719 records. After structured screening, 3687 records were retained and 2032 were not retained. Retained records spanned 1975–2026, with four published in the 1970s, 51 in the 1980s, 224 in the 1990s, 512 in the 2000s, 1136 in the 2010s, and 1759 in the 2020s. Figure 1 presents the corpus construction flow. This distribution should not be read as a direct proxy for clinical adoption, as it reflects the retrieved corpus and the search strategy used to construct it.

### 3.2. Screening Agreement

Pre-consensus agreement between the two human reviewers for the retained/not-retained decision was 99.77% (5706/5719 records), with Cohen’s κ = 0.995. After full-text adjudication, all 13 discordant records were retained. The final consensus classification contained 3687 retained records and 2032 non-retained records.

### 3.3. Outcome Domains

Pain relief or analgesic response appeared in 3550 of the 3687 retained records (96.3%) and remained the organising language of the SCS literature across the corpus. Function, quality of life, and sleep appeared in 2674 retained records (72.5%). Durability or long-term follow-up appeared in 2046 records (55.5%), and complications, revision, explantation, or device burden in 1880 records (51.0%). Trial stimulation or screening appeared in 1627 records (44.1%); patient selection, predictors, or psychological variables appeared in 1493 records (40.5%). The broad physiological-feedback aggregate appeared in 1485 records (40.3%). Paresthesia coverage or technical control appeared in 1103 records (29.9%). Patient preference, experience, or satisfaction appeared in 903 records (24.5%); economic or occupational outcomes in 839 records (22.8%); stimulation paradigm or waveform in 809 records (21.9%); and medication or opioid use in 786 records (21.3%). Table 1 summarises the outcome domains and their operational definitions.

### 3.4. Temporal Patterning of Outcome Language

**Figure 2:**
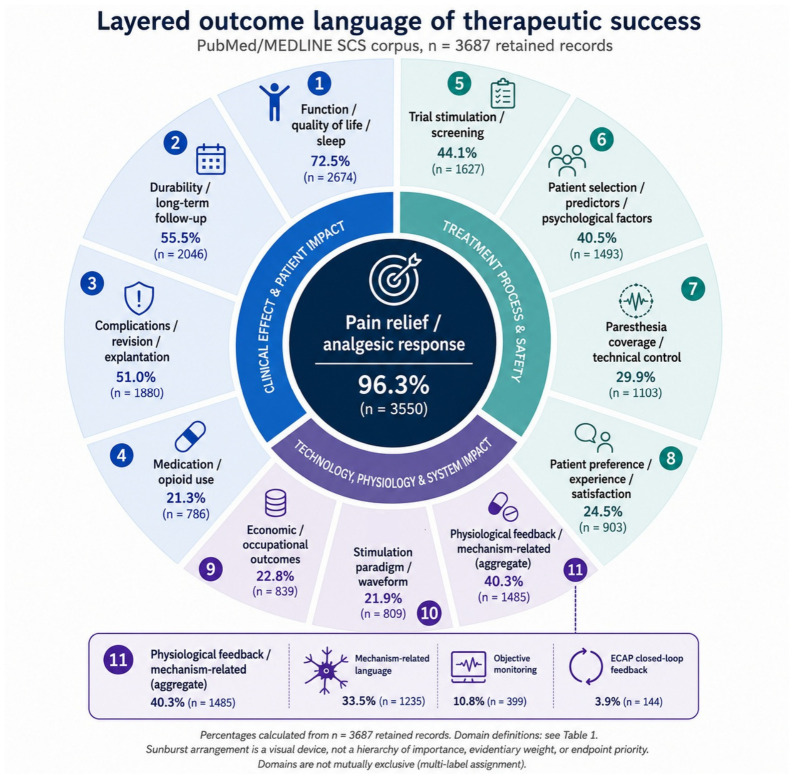
Layered outcome language of therapeutic success in the PubMed/MEDLINE spinal cord stimulation corpus. This figure is a conceptual illustration of how outcome domains are layered around an analgesic centre; it is not a quantitative or proportional representation, and segment sizes do not encode domain frequencies. Radial sunburst visualisation of non-mutually exclusive outcome domains identified across 3687 retained PubMed/MEDLINE records related to spinal cord stimulation (SCS). Pain relief or analgesic response formed the central outcome domain (3550/3687; 96.3%). Surrounding domains represent additional evaluative layers identified within the corpus, including function/quality of life/sleep, durability, complications and device burden, trial stimulation, patient selection, paresthesia coverage, patient preference, economic outcomes, stimulation paradigms, and physiological-feedback-related concepts. The physiological-feedback aggregate was secondarily separated into mechanism-related language, objective monitoring, and evoked compound action potential (ECAP)-controlled closed-loop feedback. Domains were assigned using a multi-label approach; therefore, individual records could contribute to more than one category. Segment sizes are schematic and represent conceptual layering rather than evidentiary hierarchy or proportional scaling. In particular, the relative sizes of the segments do not indicate the clinical importance of a domain and are not drawn in proportion to the frequencies reported in Table 1; the numerical domain proportions in Table 1 should be used for any quantitative comparison. Created in https://BioRender.com. Abbreviations: ECAP, evoked compound action potential; SCS, spinal cord stimulation. Figures are n/N (%). Temporal increases may reflect genuine conceptual expansion, publication-volume growth, changes in indexing, abstract length, or reporting norms, and the review cannot separate these forces.

Pain relief remained nearly universal across all decades, and the more revealing pattern was the accumulation of additional evaluative domains. Function, quality of life, and sleep appeared in 128/224 (57.1%) of retained records from the 1990s, 348/512 (68.0%) from the 2000s, 809/1136 (71.2%) from the 2010s, and 1370/1759 (77.9%) from the 2020s. Trial stimulation appeared in 34.4% of 1990s records, peaked at 47.7% in the 2000s, then stabilised at 43.8% in the 2010s and 45.1% in the 2020s. The stimulation paradigm domain showed the largest relative increase, rising from 5/224 (2.2%) in the 1990s and 17/512 (3.3%) in the 2000s to 213/1136 (18.8%) in the 2010s and 570/1759 (32.4%) in the 2020s. Economic outcomes were lower in the 1990s (19.2%) and relatively stable from the 2000s onward (22.6–23.6%). Medication use increased from 13.8% in the 1990s to 23.6% in the 2020s. Table 2 summarises the temporal patterns, and Figure 2 presents the overall layered structure of SCS outcome language across the corpus.

### 3.5. Sensitivity Check: Retained Records with Available Abstracts

Of the 3687 retained records, 3567 had abstracts available and 120 were classified using title and PubMed metadata only. To assess whether the main domain pattern depended on records without abstracts, we repeated the aggregate domain summary after excluding the 120 retained title-and-metadata-only records. The main conclusion was robust: pain relief remained the central domain (3433/3567; 96.2%), and the proportional ranking of all other domains was unchanged. The maximum absolute difference across the twelve main outcome domains between the full retained corpus and the abstract-available subset was 1.2 percentage points (durability: 55.5% vs 56.7%). Table 3 presents the full comparison. The sensitivity check does not eliminate all uncertainty related to older abstracts, non-standard reporting idioms, or publication-type heterogeneity, but it shows that the central pattern did not depend on the title-only records.

### 3.6. Sensitivity Check: Abstract Length and Temporal Trends

Because contemporary abstracts are generally longer and more structured than older ones, the temporal accumulation of outcome language could in principle reflect more text per record rather than a genuine broadening of outcome reporting. We examined this directly in the retained records with available abstracts. Abstract length did increase steadily across decades, with median word counts of 207 in the 1990s, 214 in the 2000s, 241 in the 2010s, and 260 in the 2020s. We then modelled the presence of each outcome domain as a function of its publication decade, entered it as an ordinal term, and, using logistic regression, first unadjusted and then adjusted for the natural logarithm of abstract word count. The analysis was restricted to the 3511 retained records with abstracts in decades contributing at least 200 retained records (1990s to 2020s). For the domains that grew over time, the per-decade effect was largely unchanged after adjustment. The stimulation-paradigm domain, which showed the steepest temporal rise and which the reviewers rightly singled out, had an unadjusted odds ratio of 2.68 per decade (95% CI 2.35–3.06) and an adjusted odds ratio of 2.60 (95% CI 2.28–2.97; both *p* < 0.001). Function, quality of life, and sleep moved from an unadjusted odds ratio of 1.36 (95% CI 1.26–1.47) to an adjusted value of 1.24 (95% CI 1.14–1.35), and medication or opioid use moved from 1.18 (95% CI 1.07–1.29) to 1.16 (95% CI 1.06–1.28); all three remained statistically significant. Domains that showed no temporal trend in the unadjusted model, including durability, complications, trial stimulation, patient selection, paresthesia coverage, patient preference, and economic outcomes, likewise showed no significant trend after adjustment.

A length-stratified analysis pointed in the same direction. When the retained abstracts were divided into tertiles of word count, the proportion of records using stimulation-paradigm language rose across the decades within every tertile, including the shortest abstracts (from 3.6% in the 1990s to 27.8% in the 2020s) and the longest abstracts (from 0% to 35.3%). The temporal layering of outcome language was therefore attenuated, but not explained away, by the growth in abstract length, and the principal temporal findings did not depend on that artefact. The full per-domain regression output and the length-stratified results are provided as Supplementary File S5.

### 3.7. Historical Outcome Regimes

The retained corpus does not support a simple succession of eras. Pain relief remained present across almost the entire retained literature, yet the clinical interpretation of analgesic benefit changed over time. Early PubMed-indexed records linked analgesia to trial stimulation, paresthesia, and anatomical targeting. Krainick et al. described pre-operative dorsal column stimulation as a method for estimating postoperative pain relief before implantation [1]. Hoppenstein presented percutaneous stimulation as a screening procedure before permanent implantation [2]. In these early reports, therapeutic success was not defined solely by pain reduction, but also by concordant paresthesia, anatomical targeting, and predictive trial response.

Paresthesia coverage further formalised this sensory–technical framework. Doerr et al. examined pain perception after long-term dorsal column stimulation in relation to induced paresthesia [3], while Holsheimer et al. evaluated paresthesia steering, coverage, thresholds, and therapeutic range as clinical performance measures [4]. Clinicians could map and steer paresthesia coverage as a proxy for stimulation targeting and treatment engagement.

Durability introduced an additional dimension to outcome evaluation. Long-term follow-up in amputees showed good results declining from 52.4% after two years to 39% after five years [5]. The 24-month PROCESS trial follow-up described sustained pain relief together with quality-of-life improvement, functional capacity, satisfaction, medication use, employment status, and device revision [11]. A Korean nationwide cohort study of SCS duration in patients with complex regional pain syndrome reported a median device use of approximately four years, with significant variation according to sex and insurance type, illustrating that stimulator longevity itself became a measurable outcome distinct from pain intensity alone [37]. These studies reframed success as a durable rather than purely immediate treatment effect.

Economic and social outcomes created another evaluative layer. Kumar et al. compared SCS with conventional pain therapy over five years in a cost-effectiveness analysis incorporating Oswestry Disability Index scores, medication use, and return-to-work status [8]. Later studies expanded this framework through cost-utility and real-world cost-effectiveness analyses [10,38,39,40]. These reports extended SCS evaluation into economic and health-system domains.

The trialling literature reflects a different type of historical tension. Trial stimulation remained frequent throughout the retained corpus, but increasingly became an object of evaluation itself. The TRIAL-STIM trial examined whether screening had clinical utility and cost-effectiveness [14], while accompanying patient-perspective work explored preferences regarding one-stage versus two-stage procedures [18]. The 36-month TRIAL-STIM follow-up subsequently placed trialling within a broader long-term clinical and economic framework [15]. Trial stimulation therefore persisted not only as a procedural step, but also as a subject of critical evaluation.

The multidimensional patient-reported outcome framework represented the largest expansion beyond pain intensity alone. Function, quality of life, or sleep appeared in 2674 retained records. Witkam et al. described SCS outcomes through patient expectations, experiences, satisfaction, acceptance, coping, and limitations in daily functioning [19]. Sleep became increasingly explicit in the later literature [20,21]. Together, these studies suggest that pain reduction alone was increasingly presented as an incomplete description of the broader therapeutic experience associated with SCS.

Medication use and opioid reduction formed another important outcome layer. A pooled analysis from the United Kingdom and Belgium examined changes in analgesic prescribing practices before and after SCS [22]. A SUNBURST post hoc analysis connected the waveform-specific neuromodulation literature with opioid consumption outcomes [23]. Within this literature, therapeutic success was increasingly framed not only as pain reduction, but also as reduced pharmacological dependency.

The recent physiological-feedback literature should be distinguished from broader mechanistic discussion. Mechanistic studies addressed how SCS may produce analgesia [30,31,32], whereas objective-monitoring studies evaluated whether physiological or external signals could supplement subjective outcome reporting [33]. ECAP-controlled closed-loop studies introduced a narrower device-mediated framework in which stimulation output could be adjusted according to recorded neural activation [21,34]. The EVOKE trial belongs to this third category, describing a system capable of recording evoked compound action potentials during everyday use and adjusting stimulation accordingly [34]. This shift introduced physiological feedback directly into treatment optimisation and outcome evaluation. These three uses share a vocabulary but assign the physiological signal three different roles—explanation, corroboration, and real-time control—as summarised in Table 4. The same evoked compound action potential can therefore explain a treatment, corroborate it, or steer it, and only in the last case does the physiological reading become part of the therapy rather than a description of it; reading the three as one category obscures the distinct evidentiary claim each body of work is entitled to make.

Across these historical frameworks, the dominant pattern was cumulative rather than substitutive. Pain relief remained central throughout the corpus, but the surrounding literature increasingly incorporated additional domains including durability, patient selection, device burden, quality of life, sleep, medication use, patient preference, economic impact, and physiological feedback. Success did not become multidimensional because pain ceased to matter. Rather, the literature increasingly framed pain relief as an incomplete description of the broader clinical and therapeutic context of SCS.

## 4. Discussion

If this review has one organising claim, it is this: as outcome reporting in spinal cord stimulation has broadened, the more revealing change is qualitative rather than quantitative. The corpus shifts the question from whether more outcomes exist to what kind of claim each outcome is allowed to make. Pain intensity records an analgesic effect; durability asks whether that effect survives ordinary time; and revision, explantation, and reprogramming describe the cost of keeping an implanted system clinically useful. Medication use belongs to a different register again, because it asks whether neuromodulation changes therapeutic dependency rather than pain perception alone. ECAP-controlled feedback adds a still narrower claim, namely that stimulation can be regulated through a device-recorded physiological signal. These domains sit beside one another but do not say the same thing.

The numerical pattern matters more than a simple count of domains. Pain relief remained almost universal across the decades, appearing in 221/224 records (98.7%) in the 1990s, 504/512 (98.4%) in the 2000s, 1084/1136 (95.4%) in the 2010s, and 1690/1759 (96.1%) in the 2020s. What changed was the surrounding vocabulary. Function, quality of life, and sleep rose from 57.1% in the 1990s to 77.9% in the 2020s, while stimulation paradigm moved from a marginal signal (2.2%) to a visible domain (32.4%). Figure 2 should be read in that sense, not as a hierarchy but as a record of accretion around an analgesic centre.

This pattern has a direct consequence for trial design. When the claim concerns short-term analgesic efficacy, a pain-intensity responder threshold may be defensible. Long-term registries, however, need to capture durability, revision, explantation, medication change, programming burden, and patient-reported life impact. Studies of trial stimulation should not stop at predictive value if the trial itself carries procedural burden and delays implantation. Closed-loop studies face a parallel problem, as physiological control and clinical benefit may be related, but an evoked compound action potential is not a patient-centred endpoint by another name.

A separate methodological literature has reached a similar pressure point from the opposite direction. Katz et al., reporting consensus recommendations from a joint IMMPACT, Institute of Neuromodulation, and International Neuromodulation Society meeting, proposed an endpoint matrix that included physical function, emotional functioning, sleep, health-related quality of life, cost-effectiveness, work status, patient preference, and device-specific outcomes, rather than pain intensity alone [41]. Huygen et al., using data from 509 patients in the prospective international REALITY study, found that patient-reported outcome instruments for SCS clustered around four dimensions: pain intensity, physical function and disability, psychological affect, and sleep [42]. The present corpus analysis did not test those frameworks, but it shows why such frameworks became necessary.

The issue becomes sharper in real-world evidence. Once the field understands SCS as an implanted care pathway rather than a discrete analgesic procedure, device longevity, programming frequency, revision, explantation, medication trajectories, sleep, work status, and satisfaction should not be treated as peripheral variables in long-term registries and real-world evidence studies. They mark where success is negotiated after the trial and outside the controlled research visit. A registry limited to pain scores would therefore capture the centre of the outcome claim while missing much of the later clinical dispute.

Mechanism-related language requires particular restraint. Mechanistic reviews ask how SCS may produce analgesia [30,31,32]. Objective monitoring studies ask whether external or physiological signals can supplement subjective reports [33]. ECAP-controlled studies make a more specific device claim, with stimulation adjusted according to recorded neural activation during ordinary use [21,34]. Treating these as one physiological-feedback domain is useful for screening but too coarse for interpretation.

Recent *Journal of Clinical Medicine* publications illustrate the same separation of claims. A PRISMA-ScR-informed review of SCS beyond 24 months treated durability, function, opioid use, and device maintenance as distinct long-term domains [43]. A randomised crossover study of sub- and supra-perception stimulation placed waveform selection inside the outcome frame itself [44]. A retrospective machine learning study of high-frequency SCS treated response prediction as a structured selection problem rather than a purely postoperative pain measure [45]. In that context, the present review is less a departure from current SCS writing than a map of the language that has been accumulating beneath it.

Temporal interpretation remains uncertain. Three explanations are plausible and probably inseparable: a genuine widening of clinical concerns, the growth of publication volume, and changes in abstract structure, indexing, and keyword practice. A fourth force probably acts alongside these. Reporting standards have themselves evolved: structured-abstract requirements, CONSORT- and PRISMA-type expectations, and the increasing adoption of validated patient-reported outcome measures all encourage authors to state multidimensional outcomes explicitly. The consensus endpoint frameworks and core PROM sets discussed above [41,42] both reflect and reinforce this shift, so part of the temporal layering we observe may track what journals and reporting guidelines have come to expect authors to report rather than the underlying clinical reasoning alone. The 2020s contributed 1759 retained records compared with 224 from the 1990s, so later decades carry more linguistic surface area. The safer claim is narrower: within this PubMed/MEDLINE corpus, the published language of SCS success became more layered, whether because the field changed, because reporting changed, or because both moved together. The sensitivity analysis in Section 3.6 addresses one of these forces directly. After adjusting for abstract length, the increase in stimulation-paradigm, function, and medication language persisted, so the growth in abstract size does not by itself account for the temporal pattern even though it contributes to it. A further caution runs in the opposite direction. Published language and everyday clinical practice need not move in step: a domain may enter the literature well before it reshapes routine decision-making, and a concern may be acted on at the bedside long before it becomes a fixture of abstracts. The trends reported here therefore describe how SCS success has been written about, and should not be read as a direct measure of how the therapy is practised.

The main limitation is the corpus boundary. The review used a single predefined PubMed/MEDLINE strategy, so records indexed under broader neuromodulation or pain-management terms may have been missed. Some SCS-related work is also indexed primarily in engineering- or technology-oriented databases such as IEEE Xplore, or in multidisciplinary indexes such as Scopus, Embase, and Web of Science, so a single PubMed/MEDLINE strategy does not capture that literature. Screening and charting relied on titles, abstracts, keywords, and PubMed metadata and cannot reconstruct the full endpoint hierarchy of every article. Older abstracts also used less standardised idioms, including expressions such as “good results” or “satisfactory relief”, which may not map cleanly onto later outcome domains. AI-assisted classification may handle contemporary reporting conventions more fluently than older terminology. Human adjudication reviewed all 13 discordant records, and both reviewers independently verified all 611 pre-2000 records under the same classification workflow applied to the full corpus (see Section 2.5); however, a dedicated independent re-review of the pre-2000 subset was not performed, so residual under-classification from atypical historical terminology cannot be entirely excluded. Recent decades may also appear richer simply because abstracts became longer and metadata fields more informative. A content gap is also worth noting. Cognitive outcomes and adverse psychological effects were sparsely represented in the corpus: explicit cognitive terms appeared in roughly 1.8% of retained abstracts and depression-related terms in about 7.1%, and where present they more often functioned as baseline predictors or comorbidities rather than as indexed outcomes of stimulation. The near absence of cognition and of adverse psychological effects as defined success or harm domains is itself a finding; it points to an underdeveloped area of SCS outcome reporting rather than to a settled view that these dimensions are unimportant.

## 5. Conclusions

Pain relief remains the centre of SCS outcome language, appearing in 3550 of 3687 retained records, but it is increasingly accompanied by other reported outcome domains. Across the corpus, the literature also described therapeutic success through durability, paresthesia coverage, trial response, patient selection, device burden, revision, explantation, function, quality of life, sleep, medication use, patient preference, economic value, stimulation paradigm, objective monitoring, and ECAP-controlled feedback. The pattern is cumulative rather than substitutive; analgesia persists but now shares evidentiary space with the demands of a durable, implanted, technically mediated therapy. These observations describe published outcome language within this PubMed/MEDLINE corpus and should not be interpreted as evidence of uniform changes in clinical practice, institutional policy, or patient experience across healthcare systems or device platforms. This reflects which outcomes are reported rather than how success is formally defined: the corpus shows additional domains being measured and described alongside analgesia, not a documented consensus that treatment success now requires multidimensional criteria. What has changed most clearly is not the number of outcomes but the kind of claim each is asked to support.

The review is deliberately bounded. Within that boundary, one practical implication follows: a single pain-responder threshold may suit some analgesic efficacy trials, but cannot carry the burden of long-term registries, explantation studies, sleep-focused analyses, patient-preference work, cost-effectiveness models, or closed-loop stimulation trials. The older endpoint survives, but its evidentiary role has changed.

## Figures and Tables

**Table 1:** Non-mutually exclusive outcome domains in the retained PubMed/MEDLINE SCS corpus.

Outcome Domain	Operational Definition	*n* (%)	Interpretive Role
Pain relief/analgesic response	Records referring to pain intensity, analgesia, pain reduction, responder status, or symptomatic improvement attributable to SCS.	3550 (96.3)	Establishes pain relief as the persistent anchor of SCS success language.
Function/quality of life/sleep	Records referring to physical function, disability, health-related quality of life, sleep quality, or multidimensional patient-reported status.	2674 (72.5)	Shows the widening of success beyond pain intensity into daily life consequences.
Durability/long-term follow-up	Records reporting sustained benefit, waning efficacy, or follow-up beyond the trial or implantation period.	2046 (55.5)	Frames success as a temporal claim rather than an immediate analgesic response.
Complications/revision/explantation	Records addressing adverse events, lead migration, hardware failure, reoperation, revision, explantation, or maintenance burden.	1880 (51.0)	Places therapeutic success against the burden of maintaining an implanted system.
Trial stimulation/screening	Records referring to screening trials, trial-to-implant decisions, or predictive value of trial response.	1627 (44.1)	Represents success as a selection and prediction problem before permanent implantation.
Patient selection/predictors/psychological factors	Records addressing prognostic factors, psychological assessment, indication selection, or predictors of response or failure.	1493 (40.5)	Shows the shift from asking whether SCS works to asking for whom it works.
Physiological feedback/mechanism-related (aggregate)	Broad category containing mechanism-related language (*n* = 1235, 33.5%), objective monitoring (*n* = 399, 10.8%), and ECAP closed-loop feedback (*n* = 144, 3.9%).	1485 (40.3)	Interpreted through three subcategories kept conceptually distinct.
Paresthesia coverage/technical control	Records referring to paresthesia mapping, coverage, stimulation thresholds, therapeutic range, or programming.	1103 (29.9)	Captures the sensory-technical model in which success was made visible through concordant coverage.
Patient preference/experience/satisfaction	Records addressing patient satisfaction, preference, expectations, acceptance, or willingness to repeat therapy.	903 (24.5)	Reframes success as partly dependent on the patient’s lived assessment of therapy.
Economic/occupational outcomes	Records referring to cost-effectiveness, cost-utility, healthcare resource use, work status, or return to work.	839 (22.8)	Extends success into health-system and social domains.
Stimulation paradigm/waveform	Records referring to tonic, burst, high-frequency, subperception, kilohertz, or closed-loop stimulation paradigms.	809 (21.9)	Shows that success became linked to how stimulation is delivered, not only whether it is delivered.
Medication/opioid use	Records referring to analgesic use, opioid use, medication reduction, or pharmacological displacement after SCS.	786 (21.3)	Treats success as change in therapeutic dependency, not only a change in perceived pain.

Abbreviations: SCS, spinal cord stimulation; ECAP, evoked compound action potential. Percentages use the 3687 retained records as the denominator. Domains were assigned using a multi-label approach; a single record could contribute to more than one domain.

**Table 2:** Temporal distribution of outcome domains across decades (decades with >=200 retained records).

Outcome Domain	1990s n/N (%)	2000s n/N (%)	2010s n/N (%)	2020s n/N (%)	Note
Pain relief/analgesic response	221/224 (98.7)	504/512 (98.4)	1084/1136 (95.4)	1690/1759 (96.1)	Stable across all decades.
Function/QoL/sleep	128/224 (57.1)	348/512 (68.0)	809/1136 (71.2)	1370/1759 (77.9)	Largest proportional increase across the corpus.
Durability/long-term follow-up	124/224 (55.4)	283/512 (55.3)	613/1136 (54.0)	995/1759 (56.6)	Modest but consistent increase.
Complications/device burden	109/224 (48.7)	272/512 (53.1)	632/1136 (55.6)	844/1759 (48.0)	Present in approximately half of records across all decades.
Trial stimulation/screening	77/224 (34.4)	244/512 (47.7)	498/1136 (43.8)	793/1759 (45.1)	Peaked in 2000s; persistent thereafter.
Stimulation paradigm/waveform	5/224 (2.2)	17/512 (3.3)	213/1136 (18.8)	570/1759 (32.4)	Largest relative increase; a 2010s–2020s phenomenon.
Medication/opioid use	31/224 (13.8)	100/512 (19.5)	231/1136 (20.3)	415/1759 (23.6)	Gradual increase throughout.
Patient preference/experience	56/224 (25.0)	103/512 (20.1)	298/1136 (26.2)	437/1759 (24.8)	Relatively stable proportion.
Economic/occupational outcomes	43/224 (19.2)	116/512 (22.7)	259/1136 (22.8)	415/1759 (23.6)	Lower in the 1990s; relatively stable from the 2000s onward.

Abbreviations: QoL, quality of life. Percentages use the decade totals as denominators. Temporal increases may reflect genuine conceptual expansion, publication-volume growth, changes in indexing, abstract length, or reporting norms, and the review cannot separate these forces.

**Table 3:** Sensitivity check: aggregate outcome domain proportions in all retained records versus retained records with available abstracts.

Outcome Domain	All Retained n/N (%)	Abstract-Available Retained n/N (%)	|Δ| (pp)
Pain relief/analgesic response	3550/3687 (96.3)	3433/3567 (96.2)	0.1
Function/quality of life/sleep	2674/3687 (72.5)	2606/3567 (73.1)	0.6
Durability/long-term follow-up	2046/3687 (55.5)	2021/3567 (56.7)	1.2
Complications/revision/explantation/device burden	1880/3687 (51.0)	1831/3567 (51.3)	0.3
Trial stimulation/screening	1627/3687 (44.1)	1595/3567 (44.7)	0.6
Patient selection/predictors/psychological factors	1493/3687 (40.5)	1483/3567 (41.6)	1.1
Physiological feedback/mechanism-related (aggregate)	1485/3687 (40.3)	1451/3567 (40.7)	0.4
Paresthesia coverage/technical control	1103/3687 (29.9)	1091/3567 (30.6)	0.7
Patient preference/experience/satisfaction	903/3687 (24.5)	897/3567 (25.2)	0.7
Economic/occupational outcomes	839/3687 (22.8)	822/3567 (23.1)	0.3
Stimulation paradigm/waveform	809/3687 (21.9)	773/3567 (21.7)	0.2
Medication/opioid use	786/3687 (21.3)	782/3567 (21.9)	0.6

Abbreviations: pp, percentage points. The abstract-available analysis excludes 120 retained records classified from title and PubMed metadata only. Denominators differ from total retained (*n* = 3687) because 1 retained record lacked a usable publication-year field but had an available abstract.

**Table 4:** Conceptual distinction between the three physiological-feedback subcategories identified in the corpus.

*n* (%)	Representative Literature	Underlying Evidentiary Claim	Role of the Physiological Signal	Subcategory
1235 (33.5)	Linderoth & Foreman [30]; Oakley & Prager [31]; Sdrulla et al. [32]	The signal explains the treatment effect	Explains why analgesia may occur	Mechanism-related language
399 (10.8)	Lange et al. [33]	The signal is evidence that the effect is present	Corroborates that benefit is present, as a correlate placed alongside patient-reported relief	Objective monitoring
144 (3.9)	Mekhail et al. (EVOKE) [34]; Costandi et al. [21]	The signal is an input that becomes part of the therapy	Governs stimulation delivery in real time	ECAP-controlled closed-loop feedback

Abbreviations: ECAP, evoked compound action potential. Percentages use the 3687 retained records as the denominator; the three subcategories are non-mutually exclusive.

## Data Availability

The PubMed/MEDLINE corpus, screening classification output, and reviewer-level verification/adjudication file are provided as Supplementary Files S1, S3, and S4. The retrieval script is provided as Supplementary File S2. The abstract-length sensitivity analysis output is provided as Supplementary File S5.

## Supplementary Materials

The following supporting information can be downloaded at: https://www.mdpi.com/article/10.3390/jcm15135216/s1, Supplementary File S1: PubMed/MEDLINE CSV corpus retrieved on 23 May 2026; Supplementary File S2: Anonymised Python (version 3.9.6) retrieval script using NCBI Entrez through Biopython; Supplementary File S3: Title-and-abstract screening classification output; Supplementary File S4: Reviewer-level verification and adjudication file; Supplementary File S5: Abstract-length sensitivity analysis output (per-domain logistic regression with and without adjustment for abstract length, a length-by-decade word-count summary, and length-stratified stimulation-paradigm results); Supplementary File S6: Graphical summary of the review workflow (retrieval, screening, thematic charting, and narrative synthesis). No individual patient-level clinical data are included.

## Abbreviations

The following abbreviations are used in this manuscript:

ECAP	Evoked compound action potential
IMMPACT	Initiative on Methods, Measurement, and Pain Assessment in Clinical Trials
PRISMA-ScR	Preferred Reporting Items for Systematic Reviews and Meta-Analyses extension for Scoping Reviews
SCS	Spinal cord stimulation
