# Supplementary material for: From Pain Relief to Multidimensional Outcomes: A Structured Narrative Review of Success Language in a PubMed/MEDLINE Spinal Cord Stimulation Corpus
Source: J Clin Med. 2026 Jul 3;15(13):5216. doi: 10.3390/jcm15135216 (PMC13362755; doi:10.3390/jcm15135216)
Supplement: Supplementary file 1 [file jcm-15-05216-s001.zip › Supplementary File S2_anonymised_Python_retrieval_script.pdf]

Supplementary File 2. Anonymised Python retrieval script

```
#!/usr/bin/env python3
"""
Supplementary File 2. Anonymised Python retrieval script.
Retrieves PubMed/MEDLINE XML metadata for spinal cord stimulation records using NCBI Entrez via Biopython.
The Entrez e-mail address is anonymised for manuscript submission.
"""

from Bio import Entrez
import time
import csv
import xml.etree.ElementTree as ET

Entrez.email = "anonymous@example.com"  # anonymised
SEARCH_QUERY = '("Spinal Cord Stimulation"[Mesh] OR "Spinal Cord Stimulation"[Title/Abstract])'
BATCH_SIZE = 250
SLEEP_SECONDS = 0.5
OUTPUT_CSV = "pubmed_spinal_cord_stimulation_full.csv"


def search_pubmed(query):
    handle = Entrez.esearch(db="pubmed", term=query, retmax=100000, usehistory="y")
    record = Entrez.read(handle)
    handle.close()
    return record["IdList"]


def fetch_xml(pmids):
    handle = Entrez.efetch(db="pubmed", id=",".join(pmids), rettype="xml", retmode="xml")
    xml_data = handle.read()
    handle.close()
    return xml_data


def safe_text(node):
    return "" if node is None or node.text is None else node.text.strip()


def parse_article(article):
    medline = article.find("MedlineCitation")
    pubmed = article.find("PubmedData")
    pmid = safe_text(medline.find("PMID")) if medline is not None else ""
    article_node = medline.find("Article") if medline is not None else None
```

```
title = safe_text(article_node.find("ArticleTitle")) if article_node is not None else ""
abstract_parts = []
if article_node is not None:
    abstract = article_node.find("Abstract")
    if abstract is not None:
        for part in abstract.findall("AbstractText"):
            label = part.attrib.get("Label")
            text = "".join(part.itertext()).strip()
            if label:
                abstract_parts.append(f"{label}: {text}")
            else:
                abstract_parts.append(text)
abstract_text = " ".join(abstract_parts)
journal = ""
journal_abbrev = ""
issn = ""
year = ""
month = ""
volume = ""
issue = ""
if article_node is not None:
    journal_node = article_node.find("Journal")
    if journal_node is not None:
        journal = safe_text(journal_node.find("Title"))
        journal_abbrev = safe_text(journal_node.find("ISOAbbreviation"))
        issn = safe_text(journal_node.find("ISSN"))
        ji = journal_node.find("JournalIssue")
        if ji is not None:
            volume = safe_text(ji.find("Volume"))
            issue = safe_text(ji.find("Issue"))
            pd = ji.find("PubDate")
            if pd is not None:
                year = safe_text(pd.find("Year"))
                month = safe_text(pd.find("Month"))
doi = ""
if pubmed is not None:
    for aid in pubmed.findall("ArticleIdList/ArticleId"):
        if aid.attrib.get("IdType") == "doi":
            doi = aid.text or ""
publication_types = []
if article_node is not None:
    for pt in article_node.findall("PublicationTypeList/PublicationType"):
        publication_types.append(pt.text or "")
languages = []
if article_node is not None:
    for lang in article_node.findall("Language"):
```

```
        languages.append(lang.text or "")
authors = []
affiliations = []
if article_node is not None:
    for author in article_node.findall("AuthorList/Author"):
        last = safe_text(author.find("LastName"))
        initials = safe_text(author.find("Initials"))
        if last or initials:
            authors.append((last + " " + initials).strip())
        for aff in author.findall("AffiliationInfo/Affiliation"):
            if aff.text:
                affiliations.append(aff.text.strip())
mesh_terms = []
if medline is not None:
    for mh in medline.findall("MeshHeadingList/MeshHeading"):
        desc = mh.find("DescriptorName")
        if desc is not None and desc.text:
            mesh_terms.append(desc.text)
keywords = []
if medline is not None:
    for kw in medline.findall("KeywordList/Keyword"):
        if kw.text:
            keywords.append(kw.text.strip())
grants = []
if article_node is not None:
    for grant in article_node.findall("GrantList/Grant"):
        gid = safe_text(grant.find("GrantID"))
        agency = safe_text(grant.find("Agency"))
        grants.append(" | ".join([x for x in [gid, agency] if x]))
return {
    "PMID": pmid,
    "DOI": doi,
    "Title": title,
    "Abstract": abstract_text,
    "Journal": journal,
    "JournalAbbrev": journal_abbrev,
    "ISSN": issn,
    "Year": year,
    "Month": month,
    "Volume": volume,
    "Issue": issue,
    "PublicationTypes": "; ".join(publication_types),
    "Language": "; ".join(languages),
    "Authors": "; ".join(authors),
    "Affiliations": " || ".join(sorted(set(affiliations))),
    "MeSH": "; ".join(mesh_terms),
```

```
        "Keywords": "; ".join(keywords),
        "Grants": "; ".join(grants),
    }


def main():
    pmids = search_pubmed(SEARCH_QUERY)
    rows = []
    for start in range(0, len(pmids), BATCH_SIZE):
        batch = pmids[start:start+BATCH_SIZE]
        xml_data = fetch_xml(batch)
        root = ET.fromstring(xml_data)
        for article in root.findall("PubmedArticle"):
            rows.append(parse_article(article))
        time.sleep(SLEEP_SECONDS)
    fieldnames = list(rows[0].keys()) if rows else []
    with open(OUTPUT_CSV, "w", newline="", encoding="utf-8-sig") as f:
        writer = csv.DictWriter(f, fieldnames=fieldnames)
        writer.writeheader()
        writer.writerows(rows)


if __name__ == "__main__":
    main()
```
