# Supplementary material for: From Pain Relief to Multidimensional Outcomes: A Structured Narrative Review of Success Language in a PubMed/MEDLINE Spinal Cord Stimulation Corpus
Source: J Clin Med. 2026 Jul 3;15(13):5216. doi: 10.3390/jcm15135216 (PMC13362755; doi:10.3390/jcm15135216)
Supplement: Supplementary file 1 [file jcm-15-05216-s001.zip › Supplementary File S6_workflow_bw.pdf]

Graphical summary of the review workflow

1. Retrieval

PubMed/MEDLINE search (23 May 2026)
("Spinal Cord Stimulation"[Mesh] OR
"Spinal Cord Stimulation"[Title/Abstract])
n = 5719 records (1961–2026)

2. Title-and-abstract screening

All 5719 records screened
(5144 with abstract; 575 title/metadata only)
Independent dual human review
Pre-consensus κ = 0.995

3. Thematic charting

Retained n = 3687 (1975–2026) | Not retained n = 2032
Multi-label, non-mutually exclusive outcome domains
(clinical, therapy performance, safety/utilisation,
technology/mechanism)

4. Structured narrative synthesis

Outcome concept as the unit of analysis
Layered “success” language mapped around an
analgesic centre (pain relief 96.3%)
No meta-analysis or risk-of-bias assessment

Schematic of the structured narrative review process; not a PRISMA flow diagram.
Figures correspond to those reported in the main text and Figure 1.
